# Photodegradation Controls of Potential Toxicity of Secondary Sunscreen-Derived Microplastics and Associated Leachates

**DOI:** 10.1021/acs.est.4c12077

**Published:** 2025-03-08

**Authors:** Anqi Sun, Wen-Xiong Wang

**Affiliations:** 1 School of Energy and Environment and State Key Laboratory of Marine Pollution, City University of Hong Kong, Kowloon, Hong Kong, China; 2 Research Centre for the Oceans and Human Health, City University of Hong Kong Shenzhen Research Institute, Shenzhen 518057, China

**Keywords:** secondary microplastic, photoaging, plastic leachate, degradation, nontargeted analysis

## Abstract

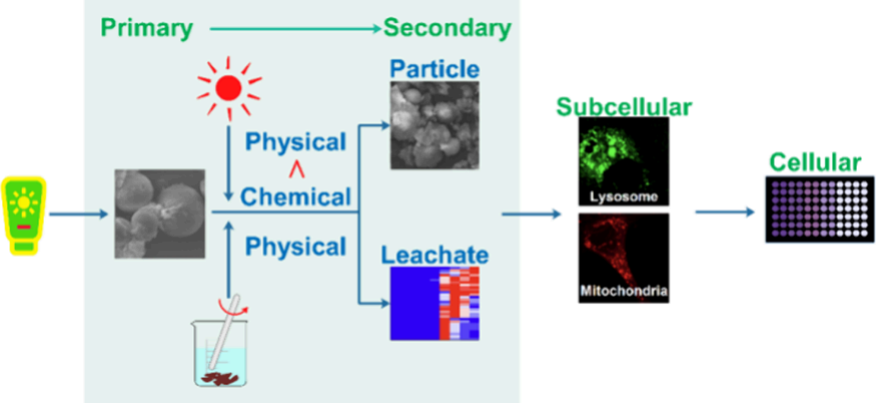

The escalating environmental concern over secondary microplastics (SMPs) stems from their physicochemical evolution from primary microplastics (PMPs), yet the contribution of varying physicochemical transformations to the ultimate environmental risks remains unknown. In this study, a photomechanical degradation process was employed to convert the primary sunscreen-derived microplastics (SDMPs) into secondary SDMPs. While mechanical degradation caused physical fragmentation, photodegradation induced both physical and chemical alterations, introducing surface oxidation, chemical bond scission, and cross-linking to the secondary SDMPs. Employing a combination of alkaline digestion and pyrolysis GC-MS techniques, it was observed that both physical fragmentation and photooxidation led to heightened intracellular sequestration of MPs. Although the bioaccumulated SDMPs could be indicated by the enlarged lysosomes and fragmented mitochondria, toxicity of secondary SDMPs at the cellular level was primarily driven by chemical transformations post-photodegradation. A nontargeted analysis employing high-resolution mass spectrometry identified 46 plastic-associated compounds in the leachate, with photodegradation-induced chemical transformations playing a crucial role in the dissociation of hydrophobic additives and oxidative conversion of leached compounds. The toxicity of the leachate was exacerbated by photodegradation, with mitochondrial fragmentation serving as the primary subcellular biomarker, indicative of leachate toxicity. This study elucidates the pivotal role of photodegradation in augmenting the cytotoxicity of secondary SDMPs, shedding light on the intricate interplay between physicochemical transformations and environmental risks.

## Abstract 2

Photodegradation heightened the environmental risks of both secondary sunscreen-derived microplastics and leachates through chemical transformations.

## Introduction

Microplastics (MPs) have been widely documented in various aquatic environments.^1^ Based on whether biological entities are involved, the environmental transformation of MPs can be broadly categorized into biotic and abiotic degradation. While both processes work collaboratively to generate secondary MPs, abiotic degradation is particularly crucial for initiating subsequent decomposition processes due to its high efficiency, especially under extreme environmental conditions.^2^ Driven by external forces stemming from interactions with environmental elements such as wind or waves, the mechanical degradation is inevitable during the transportation of MPs, ultimately culminating in the fragmentation of MPs.^3^ MPs with low fracture strain are more prone to breaking under mechanical stress, resulting in secondary MPs with smaller size, irregular shapes, and increased surface area.^4^ In an aquatic environment, fragmented MPs with a smaller size tend to have higher mobility and movement potential at both horizontal and vertical scales.^5^ In contrast to mechanical degradation, photodegradation involves more substantial chemical transformations catalyzed by sunlight. Resulting from photodegradation, MPs undergo oxidative chain scission along with radical cross-linking, yielding secondary MPs with smaller size, irregular shapes, heightened surface oxidation, and altered chemical compositions.^6^ Besides the high mobility of MPs induced by their smaller sizes, surface characteristics including the higher hydrophilicity and negative surface potentials facilitate the transportation of MPs in the soil-groundwater environment.^7^ In a real environmental scenario, multiple degradation pathways interplay to shape the physicochemical attributes of secondary MPs, significantly intertwining with their subsequent ecological risks.

Various prevalent perspectives exist regarding the physicochemical attributes contributing to their associated toxicity. The reduced size of secondary MPs augments their bioaccumulation by easing their transportation from the digestive system to the entire body, facilitating subsequent cellular uptake.^8^ Furthermore, the pronounced affinity of secondary MPs toward tissues and cells arises from their rough surfaces and serrated edges, extending their residence time and potential toxicity.^9,10^ Apart from these physical attributes from both mechanical and photodegradation processes, the emergence of oxygen-containing groups is a distinctive feature of photochemical transformation. Through the establishment of hydrogen bonding and electrostatic interactions between oxidized functional groups and biomolecules, photodegraded secondary MPs are more prone to associate with cells, impeding the intake of nutrients.^11^ Additionally, UV energy stands out as a primary driver in the creation of environmentally persistent free radicals on the surfaces of secondary MPs, triggering heightened oxidative stress in biological systems.^12^ While considerable studies have correlated the physicochemical characteristics of secondary MPs with their toxicity, there is a dearth of research focusing on pinpointing the most dominant property and its corresponding degradation pathway. Such investigations are crucial for understanding the downstream ecological risks of MPs post transformation in real-world environments.

Furthermore, plastic additives varying in different proportions (0.05–70% w/w) are weakly bounded to MPs during plastic production, rendering them susceptible to release and potentially misleading the toxicity assessment of MPs themselves.^13,14^ Given the generally biologically inert nature of most MPs, focusing solely on the toxicity of the plastics may reveal toxicity mechanisms akin to those of other bioinert materials like nanogold or silica, thereby overlooking the unique characteristic of MPs as a blend of particulate and organic contaminants. Similar to plastic particles, plastic leachates also undergo a series of abiotic transformations in the environment, resulting in a more intricate mixture with potentially harmful effects.^15,16^ Nevertheless, study on the toxicity of plastic leachates postenvironmental transformations remains limited. There is currently a notable dearth of evidence comparing plastic particles and leachate on their respective biological targets and contribution to toxicity.

The annual leakage of MPs from personal care products has reached 57 kt, potentially causing ecotoxicity due to their originally small size (<0.5 mm).^17^ In the present study, the sunscreen-derived MPs (SDMPs) were isolated to undergo the photomechanical degradation. To make the results more pronounced within a short-term experiment, coexisted *n*ZnO were introduced to accelerate the photodegradation of SDMPs, with both MPs and plastic leachate being collected for analysis. The morphology, surface chemistry, and chemical composition of degraded MPs were characterized to explain the bioaccumulation and cytotoxicity of secondary MPs. A nontargeted analysis of the leachate was conducted using a high-resolution mass spectrometry system, and the effect of degradation on cytotoxicity was subsequently revealed. This study aims to reveal the intricate interplay between physicochemical alterations and environmental risks of secondary SDMPs, highlighting the pivotal role of light and elucidating the potential mechanisms behind cytotoxic effects.

## Materials and Methods

### Photomechanical Degradation of SDMP

Considering the considerable attention given to intentionally manufactured MPs in cosmetics,^18^ we opted for commercially available sunscreens (SPF50+) to elevate the practical significance of this study. As a primary inorganic UV filter in sunscreen, *n*ZnO constituted up to 25% of the sunscreen,^19^ coexisting with MPs and accelerating photooxidation of MPs by generating hydroxyl radicals.^20^ To isolate the particulate mixture of SDMPs and *n*ZnO from sunscreens, a series of solvents with different polarities were applied to dissolve other ingredients like solvent, emollient, and surfactant in a sunscreen product. A total of 4 g of sunscreen was sequentially dissolved in 40 mL of hexane, 2-propanol, ethanol, and ultrapure water. Sonication was utilized to ensure complete dissolution of the sunscreen, with each solvent being centrifugally removed before the next solvent was added. The ZnO–SDMP mixture (0.5 g in 100 mL of ultrapure water) was added in a quartz photoreactor with continuously vigorous stirring at 1000 rpm to simulate the mechanical degradation (group M in Figure S1). An artificial sunlight lamp (OSRAM, ULTRA VITALUX 300 W, UVA-13.6 and UVB-3 W/m^2^) was applied to simulate the sunlight, and a water-cooling system was applied to avoid the high temperature during the photomechanical degradation (Figure S1). The samples were withdrawn from the reactors at predetermined intervals (12, 36, 60, and 84 h), followed by the collection of both the secondary solid mixture and leachate through a 0.22 μm membrane. We specifically focused on the mixture of MPs as they coexisted in a product. After being dried overnight (50 °C), *n*ZnO was removed by nonoxiding acid (37% hydrochloric acid) to extract secondary SDMP,^20^ which was dried at 50 °C and weighed before further experiment. To prepare SDMP under more intense mechanical degradation with a completely fractured shape, SDMP was first extracted from the ZnO–SDMP mixture by acid, followed by continuous milling in a ceramic pestle for 1 h (the group M+). The secondary SDMP (M+) and the according leachate during 12 h of stirring in the quartz photoreactor were respectively collected and preserved at 4 °C until further use.

### Characterization of the Solid SDMP

The morphology of SDMP was determined by SEM (Zeiss EVO MA10, Germany) after gold sputtering by a sputter coater (10 mA for 60 s, Quorum Q150T ES). Using an FTIR Spectrometer (PE Spectrum Two), the surface chemistry of SDMP could be determined by analyzing characteristic peaks (shown in Table S1 and Figure S2) and C–H at 1420–1500 cm^–1^ was used as the reference band, as suggested in a previous study.^21^ To determine the constituents of SDMP, a Frontier Lab Pyrolyzer equipped with an Agilent 7890*A*/5975C GC-EI/CI MSD was applied. Before using pyrolysis GC-MS, a TGA/DSC (TA STD650) analyzer was first applied to determine the thermal stability of SDMP and the weight loss of 2 mg of sample from RT to 1000 °C was recorded. After that, approximately 0.5 mg of solid SDMP was weighed and transferred to a stainless-steel sample cup for pyrolysis. Methods for double-shot pyrolysis were as follows: (1) The desorption period started at 100 °C and then increased to 200 °C at a rate of 50 °C/min, maintaining at 200 °C for 1 min. (2) The pyrolysis period lasted for 0.6 min at 600 °C. After being completely pyrolyzed, the volatile organics were injected into GC-MS and the interface temperature was set at 320 °C. The temperature program for the double-shot analysis using GC-MS was as follows: (1) The oven temperature was increased from 70 to 320 °C at a rate of 20 °C/min. (2) The oven temperature was set at 50 °C for 4 min, followed by increasing to 320 °C at a rate of 20 °C/min and maintaining at this temperature for 5 min. Separation of chemicals was done on an Ultra ALLOY metal capillary column, and a full-scan mode (40–500 *m*/*z*) was applied for analysis.

### Determination of the Solid SDMP Internalized by Cells

Dermal absorption was regarded as a major internalization pathway for MPs from cosmetics and personal care items.^17^ The HaCaT cell line was applied due to its good stability and wide application in investigating human epidermal pathophysiology. Cell-based assays, when contrasted with animal experiments, present a more affordable, consistent, and ethically preferable option for studying toxicological effects.^22^ Cells were first cultured in a T75 culture flask (89% high-glucose DMEM, 10% FBS, and 1% penicillin–streptomycin), followed by the addition of secondary SDMP (M, P+M, and M+) at 50 mg/L and coculturing for 24 h. Cells were washed by PBS for three times, numbered under an optical microscope, and collected to undergo alkaline digestion which was verified to be moderate enough to isolate SDMP from the biomatrix.^10^ Briefly, cells were mixed with 10% KOH and digested at 50 °C for 24 h. To qualitatively analyze the morphology of internalized SDMP, the digested mixture was dipped onto the 0.22 μm membrane and observed by SEM (Zeiss EVO MA10, Germany) after gold sputtering by a Sputter Coater (10 mA for 60 s, Quorum Q150T ES). Quantification of SDMP in the biomatrix was based on a previous study,^23^ where the alkaline digestion was also applied to isolate plastics from animal tissues. The digested cells dispersed in 10% KOH were further added with 10 times the volume of EtOH, followed by heating at 80 °C for 30 min and centrifugation at 8000 rpm for 5 min. The precipitates were redispersed in 180 μL of ultrapure water, and 20 μL of solution was transferred to a stainless-steel sample cup for pyrolysis. The single-shot pyrolysis was applied at 600 °C for 0.6 min, followed by the GC-MS program, as mentioned above. The sim mode was used for detection of both acrylate-based copolymer (quantification at *m*/*z* = 100) and silicone-based polymer (quantification at *m*/*z* = 207).

### Characterization of the SDMP Leachate

Due to the continuous dissolution of ZnO during the photomechanical degradation, contents of Zn^2+^ and total Zn in the leachate were first quantified using a Zn^2+^ specific fluorescence probe (i.e., (9-anthrylmethyl)bis(2-pyridylmethyl)amine) and an ICP-MS (NexION 1000, PerkinElmer, USA), respectively. The total organic carbon (TOC) was then estimated by a TOC analyzer (Shimadzu TOC-V CSH, Japan). Compounds leached during polymer degradation were complicated, involving a mixture of oligomers (high boiling points) and additives (high or low boiling points). To better identify the chemical composition of the leachate, the high-boiling-point compound and low-boiling-point compound were analyzed by pyrolysis GC-MS and a Thermo Fisher Q Exactive GC hybrid quadrupole-Orbitrap mass spectrometer, respectively. To concentrate the leachate in the pyrolysis cup, the leachate was transferred to the cup and dried at 50 °C for at least 10 cycles. The data obtained by the second-step pyrolysis program at 600 °C was recorded. The leached organics were nontargeted extracted by solvent extraction using chloroform (v/v = 1) with mechanical shaking for 10 min. The extracts were evaporated to near dryness and redissolved in 100 μL of chloroform for analysis. Separation of chemicals was performed on a DB-5HT capillary column. The oven temperature was increased from 40 to 320 °C at a rate of 10 °C/min, and a full-scan mode (50–500 *m*/*z*) was applied for analysis.

### Toxicity of SDMP and Its Leachate

We specifically focused on the toxicity of the plastic mixture and its associated leachate. For the cell viability test of SDMP, cells were precultured in a 96-multiwell plate (5000 cells/well) for 12 h, followed by the addition of SDMP at 50 mg/L (a concentration that could induce significant cytotoxicity) and coculturing for 24 h. To differentiate the cytotoxicity of leached Zn^2+^ and organics, Zn at 6 mg/L (based on the highest concentration of Zn^2+^ determined in leachate) was added in the culture medium and the influence of the EDTA chelator on cytotoxicity of Zn^2+^ was investigated by coculturing at 0.3 mM. The obtained leachate was diluted 10 times (a concentration that could induce significant cytotoxicity) in culture medium containing 0.3 mM EDTA before coculturing with cells for 24 h. For the cytotoxicity assay of both SDMP and its leachate, the cell-permeated TPEN chelator at 1 μM was added to cells to explore the possible influence of Zn^2+^, which was either adsorbed on the surface of secondary SDMP or not removed by the EDTA. Cell viability was evaluated using the MTT assay. The MTT (M6494, Invitrogen, USA) was dissolved to culture medium at 0.5 mg/mL and cultured with cells for 4 h. The absorbance at 490 nm was subsequently recorded using a microplate reader (SpectraMax M2e, USA). Determination of cytotoxicity at subcellular level was performed using a confocal microscope (Zeiss LSM 710, Germany). Cells were first cultured in a confocal dish at 150,000 cells/dish for 12 h, followed by the replacement of the culture medium to the medium containing secondary SDMP (50 mg/L) or leachate before observation. The lysosome was labeled by LysoTracker Red DND-99 (200 nM, L7528, Thermo Fisher Scientific, USA), and the mitochondrion was labeled by MitoTracker Deep Red FM (459 nM, M22426, Thermo Fisher Scientific, USA). The imaging setting for observing lysosome in cells comprised excitation at 561 nm and emission at 561–612 nm. The imaging settings for observing mitochondrion in cells comprised excitation at 640 nm and emission at 650–700 nm.

### Statistical Analysis

The statistical analysis was performed using IBM SPSS Statistics 21.0. To compare the different cellular/subcellular toxicity induced by different treatments, we used pairwise comparisons (Tukey’s HSD) by ANOVA. Before analysis, a Shapiro–Wilk test (*p* > 0.05) and a Levene’s test (*p* > 0.05) was applied to confirm the normality and equal variances of data, respectively. For data that were non-normally distributed (*p* < 0.05), the pairwise comparisons were generated by a nonparametric analysis: Kruskal–Wallis test with the *p* value adjusted by a Bonferroni adjustment. Statistical significance was indicated as follows: *p* < 0.05 (*) and *p* < 0.01 (**).

## Results and Discussion

### Constituents, Morphology, and Surface Chemistry of Secondary SDMP

Figure S3 presents the effective dispersion of particulates throughout the experiment, with the dispersed SDMP increasing to approximately 0.45 mg/mL (75% of the total) and 0.6 mg/mL (100% of the total) after undergoing mechanical and photo–mechanical degradations, respectively. During mechanical degradation, the dispersed SDMP showed a slight increase of 0.1 mg/mL from 12 to 84 h. In contrast, under photomechanical degradation, the dispersed SDMP stabilized at 100% of the total within the initial 36 h. The insignificant weight loss during degradation suggested that the majority of SDMP remained in particulate form under the specified degradation conditions. Previous study showed the obvious weight loss (>15%) of petroleum-based MPs (i.e., PP and PE) in the presence of a photocatalyst after 5 days of irradiation.^24^ Given the widespread use of antioxidants in sunscreen products,^25^ the antioxidants associated with SDMP may have partially impeded its radical-induced photodegradation.

**Figure 1:**
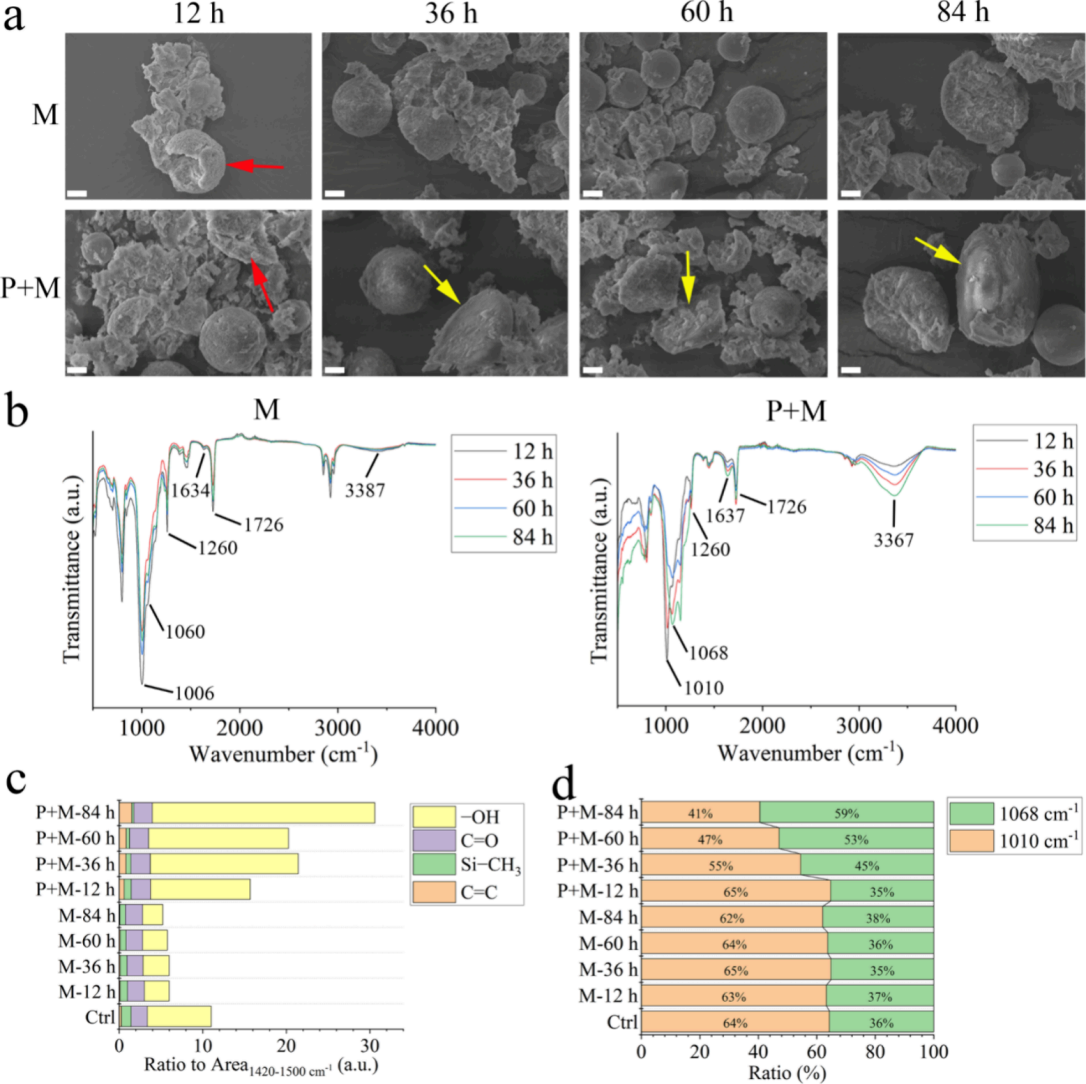
Morphology and surface chemistry of secondary SDMP. (a) Morphological change of SDMP. (b) FTIR spectra of SDMP. (c) Chemical bond index calculated from FTIR spectra. (d) Deformation of the Si–O–Si bond. The red arrow and yellow arrow in Figure 1a denote the fractured EMA-MMA (rough-surfaced) and fractured HSP (smooth-surfaced), respectively. Scale bar: 2 μm.

Figure S4 illustrates that the thermal degradation of SDMP commenced above 200 °C, with the highest degradation rate occurring between 300 and 400 °C, and near completion at 600 °C. Analysis of the volatile organics collected at 600 °C revealed that major fragments produced during thermal degradation of SDMP included methyl methacrylate, ethane-1,2-diyl bis(2-methylacrylate), and D3–D8 cyclosiloxanes (as shown in Figure S5 and Table S2). According to the pyrogram of standard polymers,^26^ the primary components of SDMP were identified as the copolymer of ethane-1,2-diyl bis(2-methylacrylate) (EMA) and methyl methacrylate (MMA) as building blocks (EMA-MMA, indicated by the red arrow in Figure S6), and the hybrid silicone powder (HSP, indicated by the yellow arrow in Figure S6). The initial SDMP mainly consisted of uniform microbeads with either smooth or rough surfaces, ranging in diameter from 4 to 12 μm (Figures S6 and S7). While HSP exhibited complete morphology during the 84 h mechanical degradation, EMA-MMA began to undergo mechanical rupture within the initial 12 h (red arrow in Figure 1a), resulting in a reduced size by 20% (Figure S7). The HSP was known for its low hardness, with its measured shore hardness was less than half of poly MMA at room temperature.^27^ Compared with EMA-MMA, the HSP was, therefore, more flexible and deformable under mechanical stress, leading to its complete morphology during mechanical degradation. Both EMA-MMA and HSP were fractured, and their sizes were reduced by 66% after intense mechanical milling (Figures S7 and S8), indicating that low hardness could only inhibit mechanical stress to a moderate extent. Under photoirradiation, HSP became susceptible to moderate mechanical forces, as evidenced by the fractured HSP during the initial 36 h of the photomechanical degradation (yellow arrow in Figure 1a). It was also noticed that larger HSP microbeads were found to be more fragile during this degradation process, with most cracked HSP appearing in larger sizes, while smaller ones remained intact. Earlier study also showed that promoted surface cracking of polyethylene was induced during the aging process,^28^ consistent with the more shrinkage of particle size (by 35%) after 84 h of photoaging in this study. The chemical modifications associated with photodegradation were correlated with the resulting physical properties of MPs, as depicted in Figure 1b–d.

The absorption band of carbonyl species, a primary degradation product of polymer oxidation, is commonly utilized to monitor the degradation process.^21^ In comparison to mechanical degradation, photodegradation intensified the surface oxidation of SDMP, as evidenced by a slight 1.2 increase in the carbonyl ratio (as shown in Figure 1c). The •OH radicals generated from the photocatalyst (ZnO in this study) were highly oxidizing species that promoted the formation of carbonyl species through hydrogen atom abstraction.^29^ The enhanced presence of hydroxyl species by 4–11 times in the presence of photodegradation was another indicator of oxidation (Figure 1c). Photoirradiation induced random homolytic scission of the main-chain C–C bond of poly MMA, leading to the formation of polymeric alcohols in the presence of air.^30^ Consistent with the photoinduced main-chain cleavage of EMA-MMA, more vinyl groups appeared post photodegradation, which was previously verified to indicate the chain scission and end-chain depolymerization.^31^ The presence of unsaturated C=C bonds lead to reduced stability against photodegradation, further promoting the polymer backbone to break down into smaller fragments.^32^ Compared with the bond energy of Si–C (318 kJ/mol), the polarized Si–O bond holds a larger bond energy at 452 kJ/mol, which is reinforced to 569 kJ/mol in the presence of methyl groups (i.e., Me_3_Si–O–SiMe_3_).^33^ Different from the photoresist Si–O bond, chemical bonds including Si–C and C–H in the side chain could break and form free radicals. The Si–CH_3_ bond gradually diminished, as shown in Figure 1b,c, with less than 25% remaining after 84 h of photomechanical degradation, whereas approximately 60% of the Si–CH_3_ bond remained intact after mechanical degradation. Following the breaking of the Si–CH_3_ bond, photogenerated radicals can react with oxygen in the air to form hydroxyl species in the side chain of silicones,^34^ partially contributing to the previously described enhanced hydroxyl index (Figure 1c). Despite the inherent stability of the Si–O–Si main chain, an increased absorbance of Si–O–Si at higher wavenumbers was observed in the presence of photodegradation (Figure 1d), indicating a higher cross-linking degree of the silicone network.^35^ It was reported that photodegradation could lead to the dominant cross-linking reaction over chain scission and increase the molecular weight of silicone polymers.^36^ The photocleaved free radicals in side chains could thus be prone to undergoing a cross-linking reaction and connecting the neighboring polymer chains in HSP.

**Figure 2:**
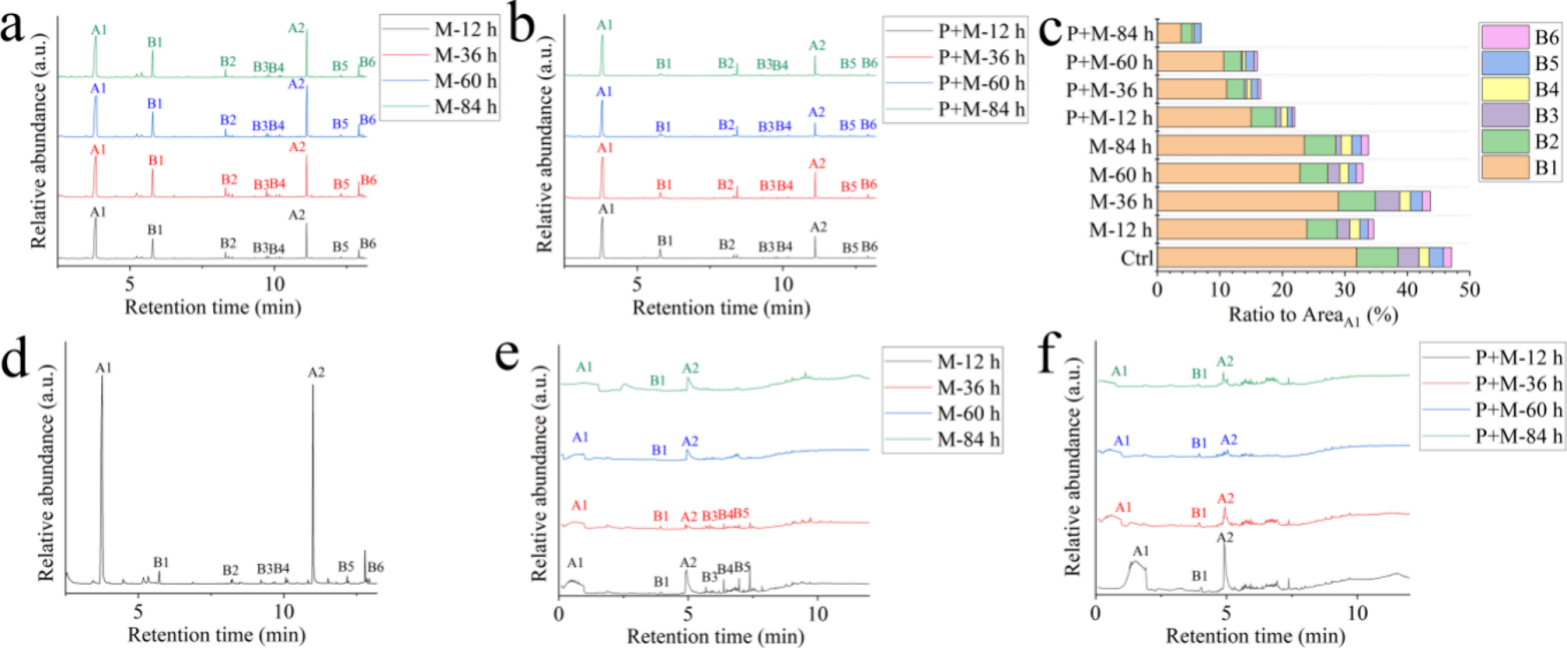
Constituents of SDMP. (a) Characteristic peaks of mechanically degraded SDMP. (b) Characteristic peaks of photomechanically degraded SDMP. (c) Pyrolysis fragments relating to HSP. (d) Characteristic peak of SDMP after intense mechanical milling. (e) Chemicals associated with mechanically degraded SDMP. (f) Chemicals associated with photomechanically degraded SDMP.

Figure 2a,b shows the pyrolysis products of SDMP under various degradation processes, with an obviously changed peak abundance in the presence of photoirradiation. Similar to the primary SDMP, methyl methacrylate (A1), ethane-1,2-diyl bis(2-methylacrylate) (A2), and D3–D8 cyclosiloxanes (B1–B6) were also identified in secondary SDMP under mechanical degradation (Figure 2a). However, the presence of D3–D8 cyclosiloxanes, reflecting the existence of HSP, gradually decreased in secondary SDMP under photomechanical degradation (Figure 2b,c). When subjected to external forces like pyrolysis, the methyl groups in silicones facilitated the rotation of Si–O–Si backbones, which were thermally cleaved to form cyclic oligomers through Si–O scission.^33^ Photoirradiation aided in the cross-linking of macroradicals in HSP by cleaving the Si–CH_3_ bond, potentially impeding the generation of cyclosiloxanes by reducing the flexibility of Si–O–Si backbones. The decline in pyrolyzed cyclosiloxanes was also observed after intense mechanical milling (Figure 2d), stemming from the decreased Si–CH_3_ bond compared to the primary SDMP (Figure S6). High cross-linking brought high performance of physical properties and hardness in polymers,^37^ leading to the gradual fracture of HSP during the photomechanical degradation (Figure 1a).

Figure S4 shows that before the onset of thermal decomposition at 200 °C, approximately 5% weight loss of SDMP was detected. The polymer-associated matrix that was decomposed at a lower temperature than those of polymers was the mixture of oligomers, additives, and nonintentionally added substances.^38^ During the desorption period of pyrolysis (≤200 °C), the chemicals were identified as methyl methacrylate, ethane-1,2-diyl bis(2-methylacrylate), and D5–D9 cyclosiloxanes (Figure S9 and Table S3), almost consistent with the pyrolysis products of polymers determined at 600 °C. Given that the onset decomposition temperatures of both EMA-MMA and HSP polymers were above 300 °C,^36,39^ the dissociated oligomers or synthesis intermediates likely constituted the major components released below 200 °C. The particulate-associated matrix diminished progressively during both mechanical and photomechanical degradations of SDMP, with only methyl methacrylate, ethane-1,2-diyl bis(2-methylacrylate), and decamethylcyclopentasiloxane remaining after 84 h (Figure 2e,f). Compared to mechanical degradation, photodegradation even accelerated the dissociation of the matrix, particularly the cyclosiloxanes, which nearly vanished during the initial 12 h of photomechanical degradation (Figure 2f). The result was consistent with the higher abundance of mixture released from plastics under UV radiation,^40^ indicating that the presence of photoirradiation could facilitate the diffusion of the plastic-associated matrix within plastic and following dissociation into plastic leachate.

### Bioaccumulation of Secondary SDMP

**Figure 3:**
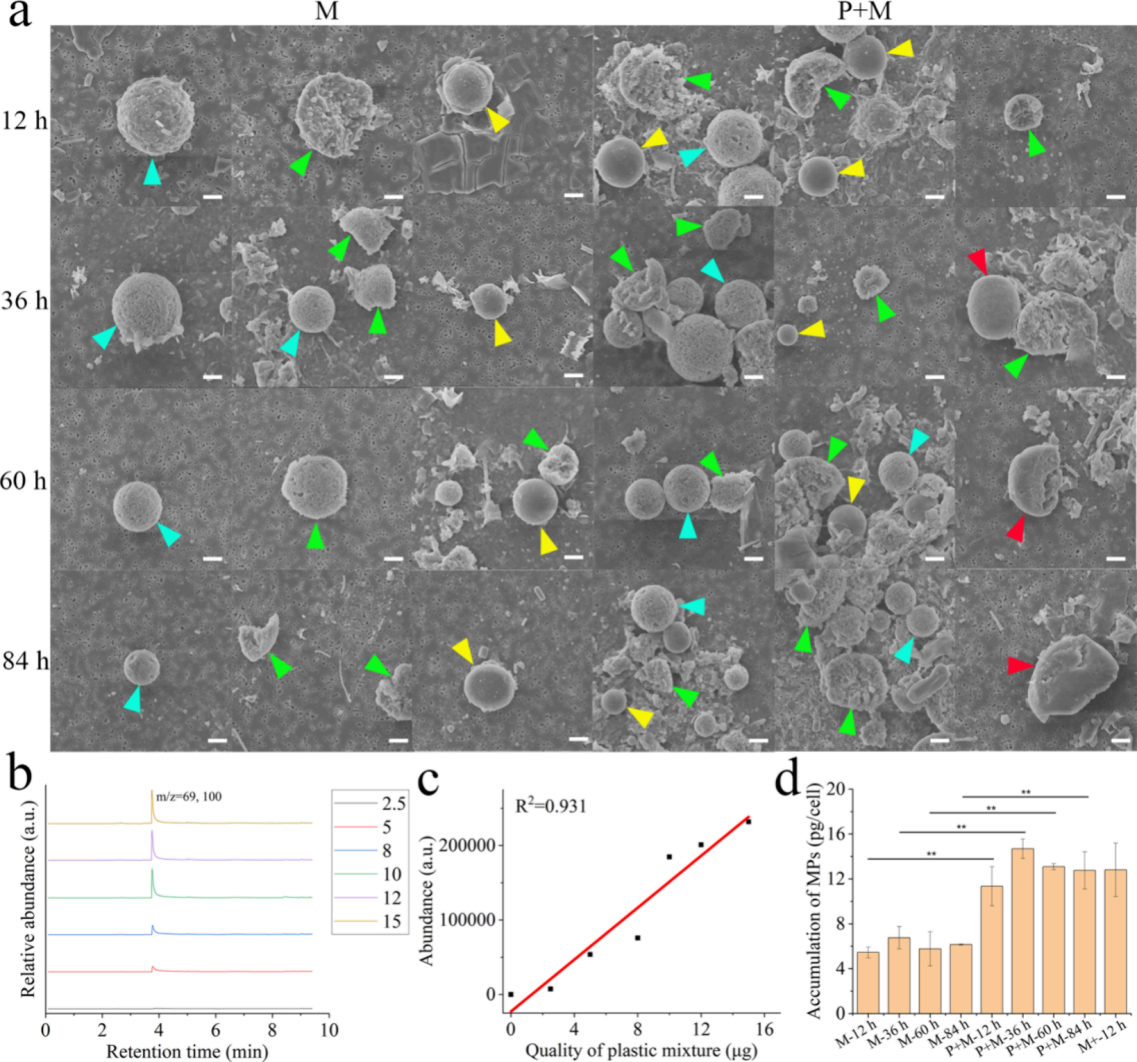
Accumulation of secondary SDMP in cells. (a) Isolation of internalized SDMP from cells. (b) The characteristic peak (*m*/*z* = 69, 100) for quantification (2.5–15 μg). (c) Standard curve for quantification (*m*/*z* = 100). (d) Accumulation of SDMP in cells. The blue arrow, green arrow, yellow arrow, and red arrow in Figure 3a denote the EMA-MMA microbead, the EMA-MMA pieces, the HSP microbead, and the HSP pieces, respectively. Scale bar: 2 μm.

During mechanical degradation, three types of secondary SDMP were internalized by cells: EMA-MMA microbeads (blue arrow), HSP microbeads (yellow arrow), and EMA-MMA pieces (green arrow) (Figure 3a). Secondary SDMP with four morphologies were isolated from cells treated with photomechanically degraded SDMP (Figure 3a), including EMA-MMA microbeads (blue arrow), HSP microbeads (yellow arrow), EMA-MMA pieces (green arrow), and HSP pieces (red arrow). This observation aligned with the morphologies of degraded SDMP before cellular uptake (Figure 1a), suggesting that secondary SDMP resulting from degradation could have an impact on cells. We also noted a consistent size of internalized SDMP across all groups (Figure S10), with intracellular MPs being smaller than their extracellular counterparts. This implies that only relatively small SDMPs in the extracellular environment may be bioavailable. Although previous reports indicated that the cellular uptake of MPs could be influenced by the material types,^41^ the nearly consistent ratio of EMA-MMA (quantified by *m*/*z* = 100) to HSP (quantified by *m*/*z* = 207) for both extracellular and internalized SDMP was noted (Figure S11). Polydimethylsiloxane and poly MMA are commonly used in microfluidic devices due to their high biocompatibility,^42^ leading to similar cellular preferences for uptake regardless of the material type.

Among all pyrolysis products identified in SDMP (Figure S12), the peak corresponding to methyl methacrylate (A_1_ peak, *m*/*z* = 100) was found to be the most abundant and, thus, considered the most sensitive component for quantifying trace amounts of SDMP in cells. Figure 3b,c shows that the integration value of the A_1_ peak (*m*/*z* = 100) correlated well with the contents of SDMP within a range of 2.5 to 15 μg (*R*^2^ = 0.931). As shown in Figure S13, the influence of the cell matrix on the intensity of the A_1_ peak (*m*/*z* = 100) was deemed negligible, enabling the utilization of *m*/*z* 100 to quantify internalized SDMP in cells following various degradation processes. Figure 3d shows that the content of mechanically degraded SDMP accumulated in cells ranging from 4 to 8 pg/cell. With the presence of photoirradiation, the accumulated SDMP increased to 11–15 pg/cell, representing more than a twofold increase compared to the accumulation under mechanical degradation alone. When compared to primary MPs, photodegraded MPs exhibited higher bioaccumulation at both the cellular and individual levels.^8,43^ To investigate the physicochemical properties of SDMP that could enhance bioaccumulation, we assessed the accumulation of intensely mechanically degraded SDMP with a completely fractured morphology. The results in Figure 3d show that the accumulated SDMP after intense mechanical milling reached 12 pg/cell, comparable to SDMP after photomechanical degradation. The size of MPs greatly affects cellular uptake, with smaller sizes being more easily ingested by cells.^44,45^ The more fragmented MPs (at approximately 2.5 μm in Figure S7) after intense mechanical degradation led to their higher cellular uptake. It is apparent that the photoinduced fragmentation of SDMP was not as significant as mechanical milling (Figure S7), yet both were equally internalized (Figure 3d). The MPs in aqueous environments tend to aggregate, impacting their fate, mobility, and bioavailability.^46^ Indicated by the enhanced carbonyl and hydroxyl index during photodegradation (Figure 1c), the hydrophobic surface of SDMP could be oxidized to become more hydrophilic, preventing aggregation in a cell culture medium and thereby enhancing subsequent cellular uptake.

### Bioeffect of Secondary SDMP at Cellular and Subcellular Levels

**Figure 4:**
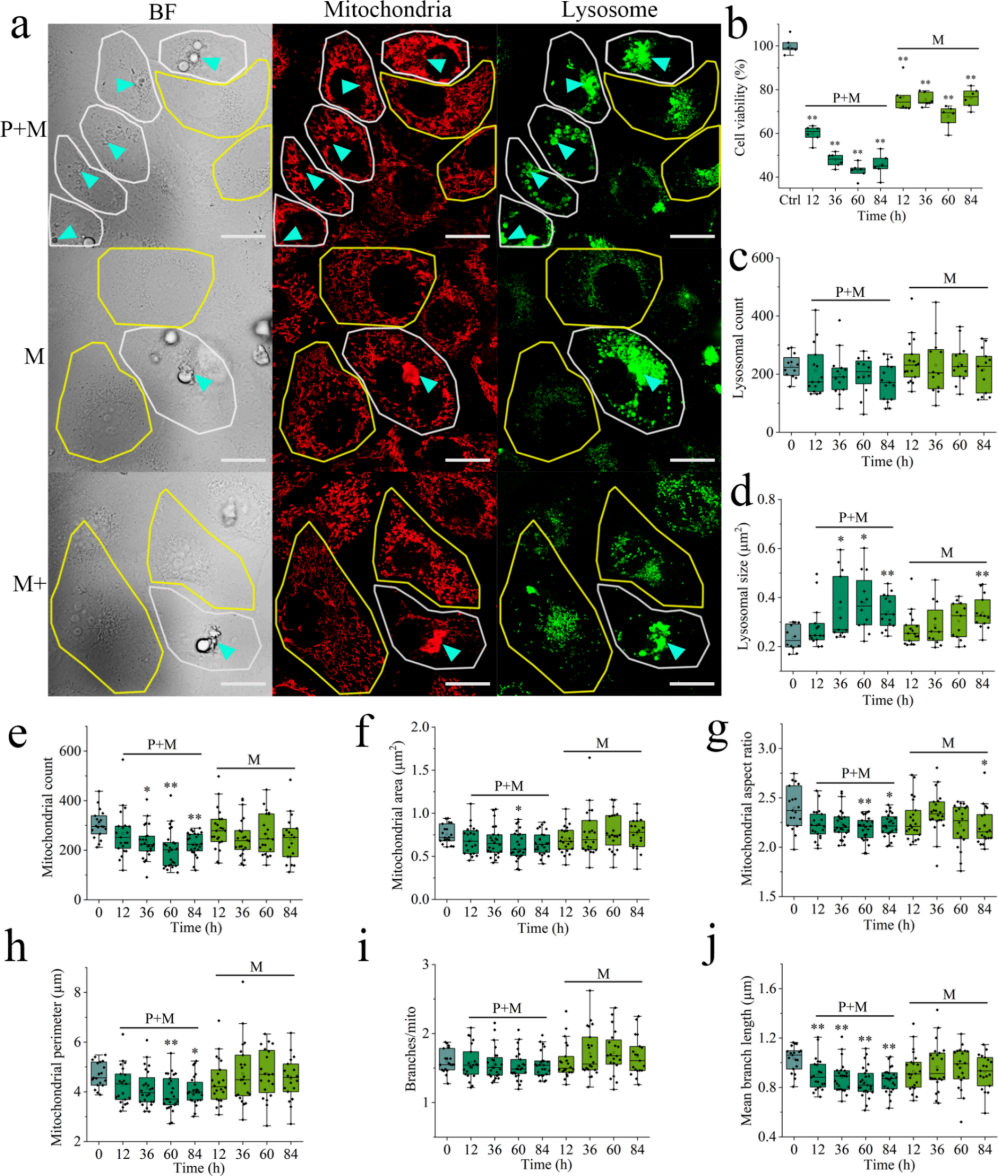
Cytotoxicity of secondary SDMP (50 mg/L). (a) Image showing the internalization of SDMP in cells. (b) Effect of SDMP on cell viability. (c, d) Effect of SDMP on lysosomal biomarkers. (e–j) Effect of SDMP on mitochondrial biomarkers. The yellow and white circles indicate the normal cells and cells internalized with SDMP (denoted by the blue arrow in Figure 4a), respectively. Scale bar: 20 μm.

Due to the heterogeneous distribution of SDMP in the culture medium, some of cells were internalized with MPs (denoted by the blue arrow in white circled cells, Figure 4a), while others remained free of MPs (in yellow circles). In contrast to the mechanically degraded SDMP, photoirradiation endowed SDMP with higher hydrophilicity, thereby facilitating a higher incidence of MPs ingestion by cells (Figure 4a). It was noted that internalized SDMP tended to cluster around the nucleus, a pattern consistent with previous studies on the ingestion of polystyrene in human cells.^47,48^ A novel endosomal route was proposed to transport proteins on the cell surface to the nucleus,^49^ potentially leading to the accumulation of SDMP-laden vesicles around the nucleus postinternalization. While the primary SDMP at 50 mg/L did not induce significant cytotoxicity (Figure S14), the presence of SDMP reduced cell viability to below 80% after 12 h of mechanical degradation (*p* < 0.01 in Figure 4b). The duration of mechanical degradation did not significantly impact the SDMP-induced loss of viability, possibly due to the stabilized chemical composition and bioaccumulation of secondary SDMP (Figures 1b, 2a, and 3d). In contrast to the moderate toxicity induced by mechanically degraded SDMP, the presence of photodegradation reduced cell viability to around 40%, with cytotoxicity increasing as degradation time was extended (Figure 4b). Although intensely milled SDMP was completely fragmented and accumulated to levels similar to that of photodegraded SDMP, the resulting cytotoxicity was lower than its photodegraded counterpart (Figure S15). This suggests that the chemical composition altered by photodegradation, rather than the morphology and bioaccumulation, primarily contributed to the photoinduced cytotoxicity of SDMP.

Subcellular biomarkers have been highlighted for their sensitivity in indicating both the presence and biological effects of particulates within cells,^45^ emphasizing the importance of analyzing cells engulfed with secondary SDMP at a subcellular level. Figure 4c,d reveals the effect of secondary SDMP on the lysosomal structure, and the lysosomal size was regarded as a more sensitive biomarker to secondary SDMP than the lysosomal count. Compared with the mechanically degraded SDMP, the presence of photodegradation notably increased the lysosomal size in cells by approximately twofold (*p* < 0.01, Figure 4d). Plastics enclosed within endosomes eventually merge with lysosomes,^50,51^ leading to lysosomal enlargement, a phenomenon consistent with the enlarged lysosomes resulting from the significant accumulation of SDMP after photomechanical degradation (Figure 3d). While the high bioaccumulation of photomechanically degraded SDMP may not be the primary reason for its cytotoxicity, the internalized SDMP could be closely linked to the increased lysosomal size, as evidenced by the enlarged lysosomes in the presence of intensely milled SDMP (Figure S16). In contrast to the even distribution of lysosomes in normal cells (Figure S17), lysosomes tended to concentrate around the nucleus in cells associated with SDMP (denoted by the blue arrow in Figure 4a). Typically, lysosomes move toward the cell periphery in nonpolarized cells, whereas perinuclear movement is linked to various lysosomal storage diseases.^52^ The accumulation of lysosomes and secondary SDMP around the cell nucleus suggests that endosome–lysosome fusion could facilitate the damage of SDMP on lysosomes and the subsequent perinuclear movement of affected lysosomes. Photodegradation is thus capable of enhancing the lysosomal toxicity of mechanically degraded SDMP by boosting its cellular uptake.

Similar to the insignificant influence of mechanically degraded SDMP on lysosomes, its effect on mitochondrial dynamics was also limited (Figure 4e–j). In contrast, the presence of photomechanically degraded SDMP altered mitochondrial morphology, leading to a decrease in the mitochondrial aspect ratio, perimeter, and mean branch length (Figure 4g,h,j). Studies have shown that mitochondrial fragmentation can be induced by high concentrations of internalized particulates,^53^ suggesting that the abnormal mitochondrial morphology observed may be attributed to the high bioaccumulation of SDMP under photoirradiation. Intense mechanical milling could similarly enhance the bioaccumulation of SDMP, leading to significant changes in parameters associated with mitochondrial fragmentation (*p* < 0.01 in Figure S16). In addition to changes in mitochondrial morphology, a decreasing trend in mitochondrial number was noted in cells exposed to photomechanically degraded SDMP (*p* < 0.05 in Figure 4e), contrasting with the insignificant mitochondrial loss observed in cells treated with mechanically degraded SDMP. Unlike the abnormal mitochondrial morphology induced by intensely milled SDMP, the associated mitochondrial loss was insignificant (Figure S16). Cells typically degrade and eliminate dysfunctional mitochondria to maintain a healthy network.^54^ While mitochondrial morphology may be distorted by highly accumulated SDMP, only photodegraded SDMP was found to induce severe mitochondrial dysfunction, prompting the cell to eliminate damaged mitochondria.

### Chemical Composition of Leachate from Secondary SDMP

**Figure 5:**
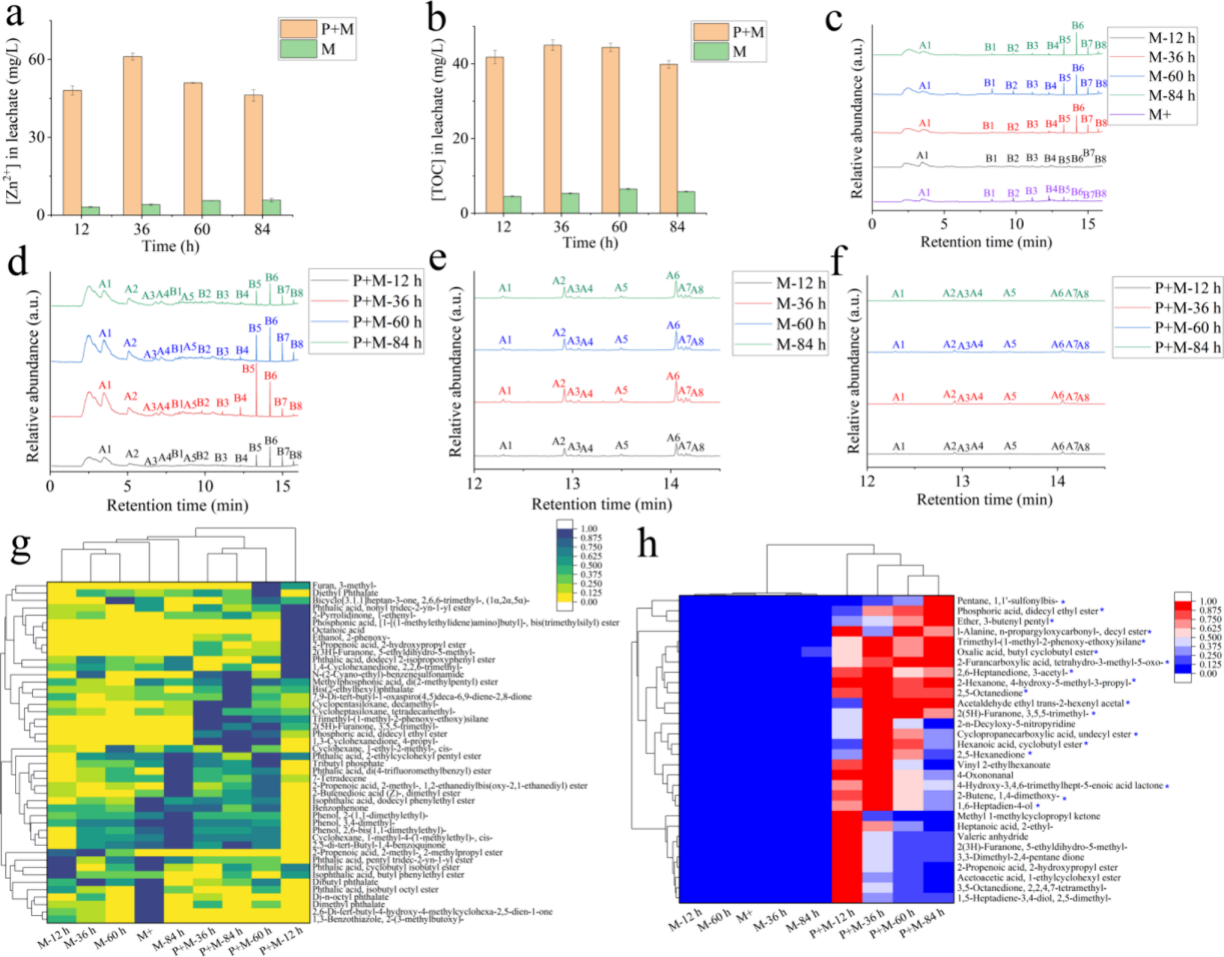
Chemical composition of the SDMP leachate. (a) Concentration of Zn^2+^ in leachate. (b) Concentration of TOC in leachate. (c, d) Characteristic peaks of pyrolysis product of SDMP leachate. (e, f) Characteristic peaks of pyrolysis product of SDMP. (g) Compounds potentially used as plastic processing aids (normalization: 0–1). (h) Compounds that enhanced by photoirradiation (fold change >5, normalization: 0–1, “*” following the compound name indicates its priority in inducing cytotoxicity).

Besides components in plastic leachate, the coexistence of *n*ZnO with SDMP resulted in the inevitable release of Zn^2+^ or dispersed *n*ZnO as coexisting components. Figure 5a illustrates that the photoirradiation promoted the dissolved Zn^2+^ by over eightfold. The enhanced dissolution was attributed to the action of photogenerated holes on the Zn–O bond, leading to the disassociation of Zn^2+^ from the surface of ZnO.^55^ Additionally, the total content of Zn was detected to be comparable to the dissolved Zn^2+^ (Figure S18), suggesting that the majority of Zn existed in ionic form and the contribution of filtrated *n*ZnO could be neglected. In addition to Zn^2+^, the presence of photodegradation also accelerated the release of TOC, with over sixfold more TOC being released compared to mechanical degradation alone (Figure 5b). Although intense mechanical milling led to a higher release of TOC compared to moderate mechanical degradation (Figure S19), the amount was still four times lower than the amount of TOC released in the presence of photodegradation. The substantial increase in TOC release aligns with findings from prior research, where leachate collected during plastic photoaging was identified as containing degradation products of MPs or additives used during plastic production.^24,56^

In Figure 5c, various cyclosiloxanes (peaks B1–B8) with different molecular weights (D4–D10 in Table S4) were leached during mechanical degradation, showing a gradual increase over time. This observation aligns with the gradual decrease in cyclosiloxanes associated with mechanically degraded SDMP (Figure 2e), indicating that oligomers or synthesis intermediates of silicones could be released as part of the leachate. Compared with the M-12 h group, the intense mechanical milling (M+ group) facilitated the dissociation of cyclosiloxanes due to its complete fracture of SDMP (Figure 5c). Although not as extensively fractured as the M+ group, photomechanically degraded SDMP leached a higher content of cyclosiloxanes (Figure 5d), particularly within the first 12 h, consistent with the disappearance of cyclosiloxanes from SDMP during the initial 12 h of degradation (Figure 2f). The desorption kinetics of organics from plastics were influenced by factors such as the particle–water partition coefficient and desorption time.^57^ Given the high surface oxidation of SDMP during photodegradation, the hydrophobic cyclosiloxanes could more easily dissociate from oxidized SDMP compared with the primary SDMP. In addition to cyclosiloxanes, elevated levels of aromatic substances were identified, including BTX aromatics (benzene, toluene, and xylene) and α-methylstyrene (peaks A1-A5 in Figure 5d). These aromatic substances could result from the breakdown of plastic additives or the pyrolysis of paraffins.^38,58^ A series of long-chain alkenes (C13–C20, peaks A1–A8 in Figure S20, Figure 5e,f, and Table S5) gradually diminished in photoaged MPs, indicating the progressive loss of paraffin to leachate. The loss of paraffin could be attributed to photodissociation from the main-chain Si–O–Si (e.g., C30–45 alkyl cetearyl dimethicone crosspolymer), which subsequently released to leachate and pyrolyzed into aromatic substances.

Using nontargeted analysis with a high-resolution mass spectrometry system, a total of 1941 features were identified in the leachate during the degradation of SDMP. The detection of a multitude of compounds cannot solely be attributed to additives used during the manufacturing of SDMP. The SDMP, due to its prolonged coexistence with other organic ingredients in sunscreens and plastic packaging bottles of sunscreens (LDPE and PP in this study), could act as carriers to absorb and transfer numerous organics, thereby increasing the complexity of the detected compounds. Referring to previously reported chemicals associated with plastic production,^15,59^ 46 compounds were further filtered (high-resolution filtering score >90) due to their known functions in polymer manufacturing (Figure 5g). These compounds included antioxidants (phenol, 2-(1,1-dimethylethyl), and benzoquinone), light stabilizers (benzophenones), plasticizers (benzenesulfonamide and phthalates), flame retardants (organophosphorus compounds), odor agents (compounds containing furan or 2-furanone), lubricants (tetradecene), and intermediates (cyclosiloxanes for silicone synthesis). Plastic additives upregulated by photodegradation included phthalate plasticizers (bis(2-ethylhexyl)phthalate, diethyl phthalate, phthalic acid, and nonyl tridec-2-yn-1-yl ester), decamethylcyclopentasiloxane, 2-hydroxypropyl methacrylate, trimethyl-(1-methyl-2-phenoxy-ethoxy)silane, organophosphorus compounds (phosphoric acid, didecyl ethyl ester, and methylphosphonic acid, di(2-methylpentyl) ester), and odor agents (3,5,5-trimethyl-2(5*H*)-furanone and 5-ethenyldihydro-5-methyl-2(3*H*)-furanone). In addition to the known additives, 30 compounds with unclear functions were upregulated by over fivefold in the presence of photoirradiation (Figure 5h). Interestingly, the abundance of compounds varied over time, with some decreasing after an initial rise, while others showed a continuous increase or remained relatively stable under irradiation (Figure 5h). It was reported that the UV irradiation increased the complexity of plastic leachate and promoted a shift toward more hydrophilic compounds.^16^ Considering that the bioavailability and toxicity of compounds could change during irradiation,^40^ the presence of photodegradation may complicate the environmental transport and subsequent biological effects of the primary leachate by changing the original chemical compositions.

### Bioeffect of Leachate at Cellular and Subcellular Levels

**Figure 6:**
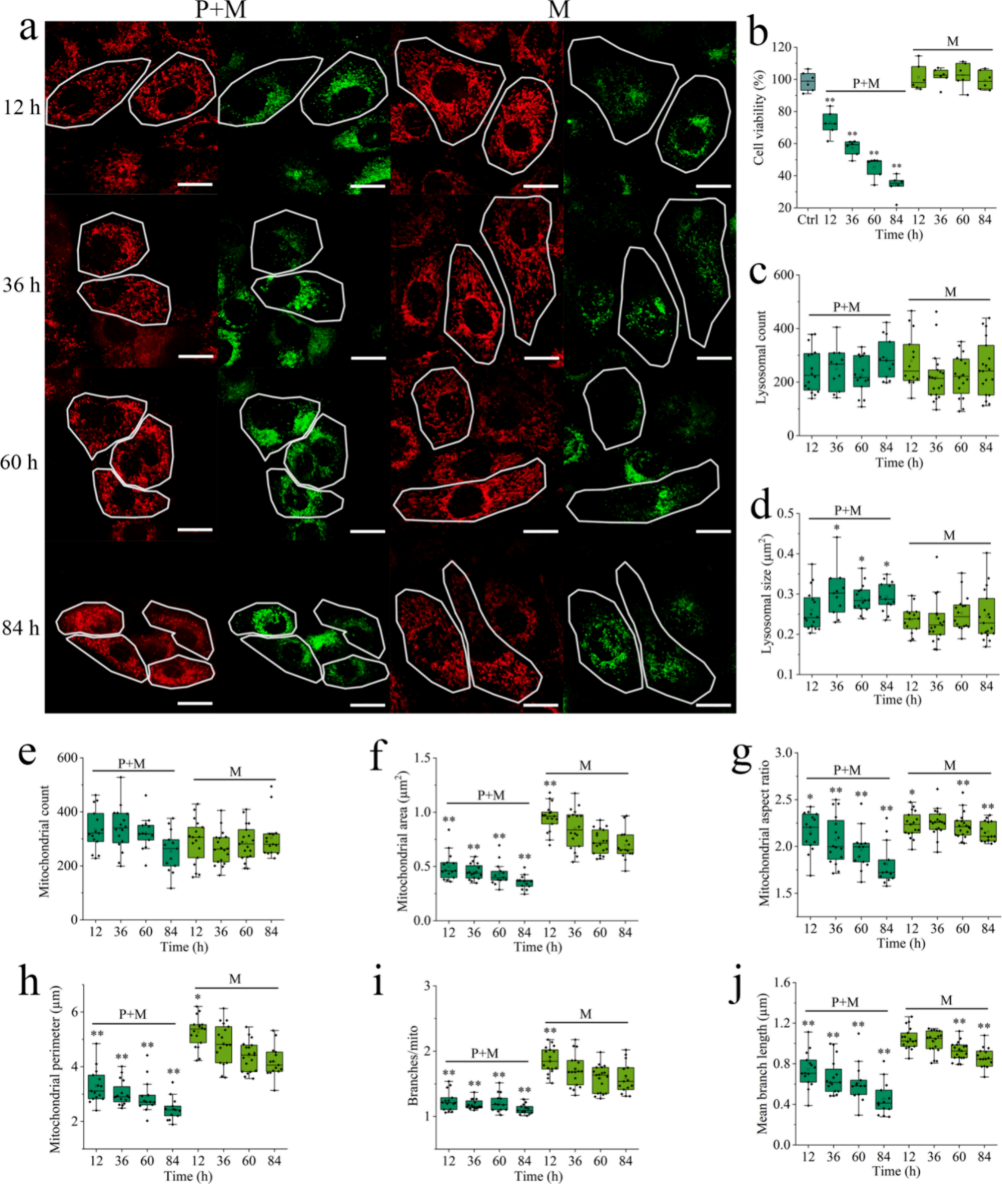
Cytotoxicity of SDMP leachate (dilution 10 times for cytotoxicity assay). (a) Image showing cells of interest (circled in white). (b) Effect of SDMP leachate on cell viability. (c, d) Effect of leachate on lysosomal biomarkers. (e–j) Effect of leachate on mitochondrial biomarkers. Scale bar: 20 μm. Control group is shown in Figure 4.

The homogeneous dispersion of leachate in the cell culture medium resulted in significant uniformity in cellular states within the same group (Figure 6a). This stands in contrast to the prior heterogeneous distribution of SDMP (Figure 4a), where even neighboring cells within the same group could manifest divergent biological responses owing to variances in intake quantities. When compared to mechanically degraded SDMP, diluted leachate from photomechanically degraded SDMP led to noticeable cell shrinkage and organelle deformation (Figure 6a). Loss of cell volume is a ubiquitous characteristic of programmed cell death.^60^ The cell apoptosis could thus be induced by the leachate of gradually photoaged SDMP, as evidenced from the substantial loss of cell viability shown in Figure 6b (*p* < 0.01). The cell viability induced by photoirradiated leachate was correlated with the photoaging time, gradually decreasing to 34% with treatment by the 84 h leachate. Incorporating Zn^2+^ did not affect cell viability (Figure S21), indicating that the leachate’s toxicity was primarily due to leached organics during photomechanical degradation. The intensely mechanical milling facilitated the leaching of organics from SDMP, including cyclosiloxanes, antioxidants, light stabilizers, and phthalate plasticizers (Figure 5c,g), which possibly led to its decreased cell viability by 12% (Figure S22, *p* < 0.05). However, its cytotoxicity was still lower than the photoirradiated leachate (12 vs 28% loss of viability), suggesting that the dissociated organics facilitated by morphological fracture was not the dominant reason for the enhanced toxicity of leachate in the presence of photoirradiation. For the abovementioned 30 compounds that were significantly upregulated by photoirradiation (>5-fold change in Figure 5h), 60% of the compounds showed high correlation to the viability loss (shown as compounds with “*” in Figure 5 h, Pearson correlation coefficient < −0.68, *p* < 0.05). Photocatalytic oxidation is expected to promote the morphological fracture and surface oxidation of SDMP, potentially facilitating the dissociation of plastic-associated organics with toxic potential. On the other hand, the photogenerated oxidative radicals were capable of mineralizing various organic pollutants,^61^ potentially reducing their end-point toxicity. The variation in compound abundance during photodegradation suggested the generation of numerous unknown oxidative intermediates with unknown toxic effects, complicating the explanation of their combined toxicity.

Figure 6c,d reveals that the leachate from mechanically degraded SDMP was nontoxic to lysosomes, whereas the leachate from photomechanically degraded SDMP slightly increased the lysosomal size by 1.3-fold (*p* < 0.05). While mechanically degraded SDMP had an insignificant effect on mitochondrial dynamics, its corresponding leachate notably influenced mitochondrial shape and branching, especially in the M-60 h and M-84 h groups (*p* < 0.01, Figure 6e–j). Interestingly, a contrary trend in mitochondrial changes was observed for the M-12 h and intensely milled M+ groups (Figure 6e–j and Figure S23), with increased area, perimeter, and branching (*p* < 0.05). Figure 5c,d indicates a slight increase in cyclosiloxanes in the leachate from these groups compared to other treatments, where cyclosiloxanes were more significantly enhanced. Although cyclosiloxanes' migration from silicone rubber was verified to induce cytotoxicity,^62^ the low-dose stress on mitochondria might have a stimulating effect known as mitohormesis.^63^ Alongside cell shrinkage, mitochondria treated with photoirradiated leachate were severely fragmented (Figure 6a), as evidenced by decreased mitochondrial size, aspect ratio, perimeter, and branching (*p* < 0.01, Figure 6e–j). Mitochondrial fragmentation typically occurs in programmed cell death,^64^ aligning with the reduced cell viability induced by leachate of photomechanically degraded SDMP. Compared to SDMP, which has limited intracellular movement due to vesicular entrapment,^65^ organics in the leachate can more easily traverse across different organelles, leading to the more significant mitochondrial toxicity of irradiated leachate than particulates.

### Environmental Implications

Given the intricate nature of environmental systems and the diverse degradation pathways of MPs, understanding the unique characteristics of these pathways and their respective contributions to the physicochemical attributes of secondary MPs is essential in understanding the plastic life cycle. This study, focusing on secondary SDMPs due to their prevalence in aquatic environments alongside ZnO catalysts, highlights the critical role of chemical transformations, particularly oxidation, in driving the cascade toxicity of SDMPs and their leachate. Despite the fact that cytotoxicity effects were observed at concentrations that may be unrealistically high in natural environments, we propose that subcellular biomarkers, such as the lysosomal size for particle presence and mitochondrial fragmentation for leachate toxicity, serve as highly sensitive indicators of environmental impact. Although microbeads added to cosmetics have been banned in at least 14 countries, polymers such as EMA-MMA and HSP as key components in SDMPs were widely used in various personal care products. Thus, the implications drawn from this research extend beyond MPs from sunscreens, offering valuable insights into predicting the environmental fate and ecological risks associated with MPs and their additives across different domains.

## Supporting Information Available

Assigned chemical bonds of the FTIR spectrum; peak assignment of SDMP by Py-GC-MS; peak assignment of chemicals associated with SDMP by Py-GC-MS; peak assignment of the leachate fragment by Py-GC-MS; peak assignment of SDMP by Py-GC-MS (RT12–14.5 min); dispersion of the ZnO-SDMP mixture during the photomechanical degradation; integrated area of FTIR spectra after baseline correction; content of dispersed SDMP during the experiment; TGA of SDMP; characteristic peak of SDMP by Py-GC-MS; morphology (left) and surface chemistry (right) of SDMP; size of MPs after degradation; morphology of the intense mechanically milled SDMP (M+ group); characteristic peak of associated chemicals on SDMP; size of ingested MPs; difference between the characteristic peaks of SDMP before and after cellular uptake; characteristic peaks of SDMP with different concentrations; effect of the cell matrix on the intensity of the quantitative peak; cell viability in the presence of primary SDMP or TPEN chelators; cell viability influenced by the presence of intense mechanically milled SDMP; subcellular effect of the intense mechanically milled SDMP; subcellular biomarkers of cells in the control group; concentration of total Zn in leachate of SDMP; concentration of TOC in 12 h leachate of intense mechanically milled SDMP; characteristic peak denoting paraffin in SDMP; effect of ionic Zn on cell viability; effect of 12 h leachate from the intense mechanically milled SDMP on cell viability; and subcellular effect of the 12 h leachate from the intense mechanically milled SDMP (PDF)
