# Supplementary material for: Photodegradation Controls of Potential Toxicity of Secondary Sunscreen-Derived Microplastics and Associated Leachates
Source: Environ Sci Technol. 2025 Mar 8;59(10):5223–36. doi: 10.1021/acs.est.4c12077 (PMC11924215; doi:10.1021/acs.est.4c12077)
Supplement: Supplementary file 1 — es4c12077_si_001.pdf [file es4c12077_si_001.pdf]

Supporting Information for

Photodegradation Controls of Potential Toxicity of Secondary Sunscreen-Derived Microplastics and Associated Leachates

Anqi Sun[1,2], Wen-Xiong Wang[1,2*]

[1]School of Energy and Environment and State Key Laboratory of Marine Pollution, City University of Hong Kong, Kowloon, Hong Kong, China

[2]Research Centre for the Oceans and Human Health, City University of Hong Kong Shenzhen Research Institute, Shenzhen 518057, China

*Corresponding author: wx.wang@cityu.edu.hk

The following are included as supporting information for this paper:
Number of pages: 12

Supplementary Figures: 23

Supplementary Tables: 5

Table S1. Assigned chemical bonds of FTIR spectrum.

Position (cm^{-1})*	Assigned configuration
1420-1500	Reference band (C-H)
1575-1695	C=C
1240-1285	$Si\text{-}CH_3$
3010-3700	O-H
1675-1765	C=O
900-1100	Si-O-Si

*Peak index=Integrated peak area/peak area$_{1420\text{-}1500\ cm\text{-}1}$

Table S2. Peak assignment of SDMP by Py-GCMS.

Label	Assignment
A1	Methyl methacrylate
A2	Ethane-1,2-diyl bis(2-methylacrylate)
B1	Hexamethylcyclotrisiloxane (D3)
B2	Octamethylcyclotetrasiloxane (D4)
B3	Dodecamethylcyclohexasiloxane (D6)
B4	Decamethylcyclopentasiloxane (D5)
B5	Tetradecamethylcycloheptasiloxane (D7)
B6	Hexadecamethylcyclooctasiloxane (D8)

Table S3. Peak assignment of chemicals associated with SDMP by Py-GCMS.

Label	Assignment
A1	Methyl methacrylate
A2	Ethane-1,2-diyl bis(2-methylacrylate)
B1	Decamethylcyclopentasiloxane (D5)
B2	Dodecamethylcyclohexasiloxane (D6)
B3	Tetradecamethylcycloheptasiloxane (D7)
B4	Hexadecamethylcyclooctasiloxane (D8)
B5	Octadecamethylcyclononasiloxane (D9)

Table S4. Peak assignment of leachate fragment by Py-GCMS.

Label	Assignment
A1	Benzene
A2	Toluene
A3	p-Xylene
A4	o-Xylene
A5	α-Methylstyrene

B1	Octamethylcyclotetrasiloxane (D4)
B2	Decamethylcyclopentasiloxane (D5)
B3	Dodecamethylcyclohexasiloxane (D6)
B4	Tetradecamethylcycloheptasiloxane (D7)
B5	Hexadecamethylcyclooctasiloxane (D8)
B6	Octadecamethylcyclononasiloxane (D9)
B7	Eicosamethylcyclodecasiloxane (D10)
B8	Unidentified (fragment of silicone rubber)

Table S5. Peak assignment of SDMP by Py-GCMS (RT12-14.5 min)

Label	Assignment
A1	1-Tridecene ($C_{13}H_{26}$)
A2	1-Tetradecene ($C_{14}H_{28}$)
A3	1-Pentadecene ($C_{15}H_{30}$)
A4	1-Hexadecene ($C_{16}H_{32}$)
A5	1-Heptadecene ($C_{17}H_{34}$)
A6	1-Octadecene ($C_{18}H_{36}$)
A7	1-Nonadecene ($C_{19}H_{38}$)
A8	1-Eicosene ($C_{20}H_{40}$)

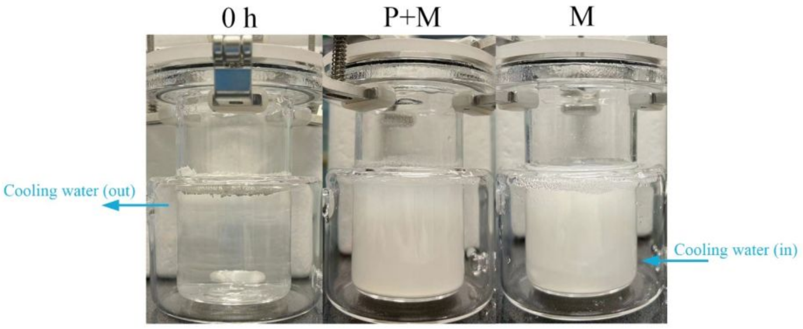

Figure S1. Dispersion of ZnO-SDMP mixture during the photo-mechanical degradation. The arrows denote the continuous input/output of the cooling tap water to the outer chamber of the photoreactor.

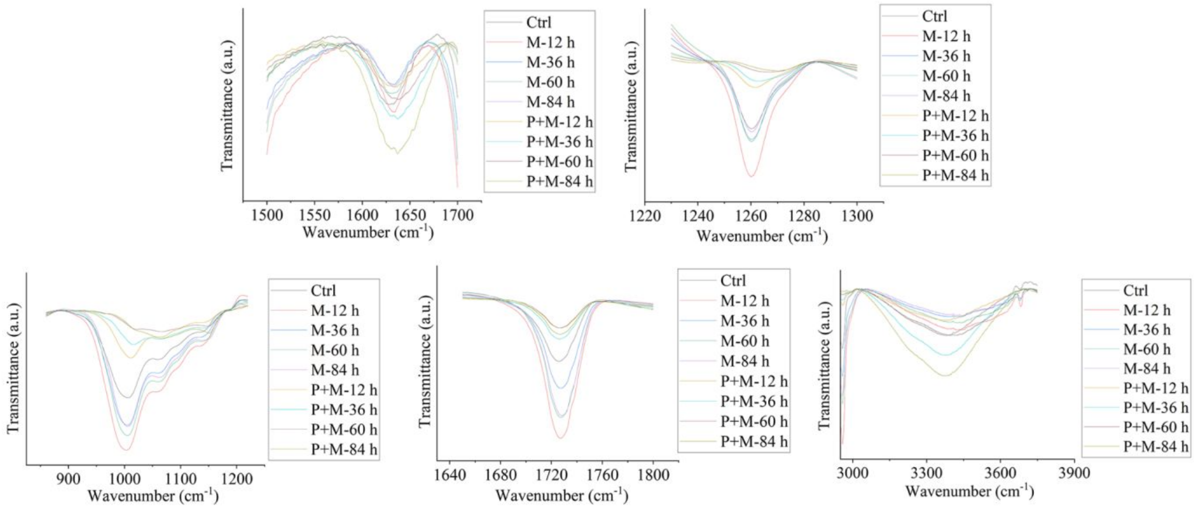

Figure S2. Integrated area of FTIR spectra after baseline correction.

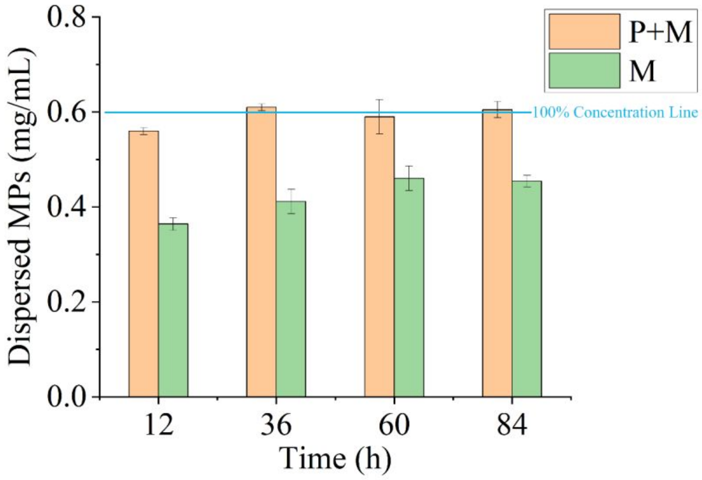

Figure S3. Content of dispersed SDMP during the experiment.

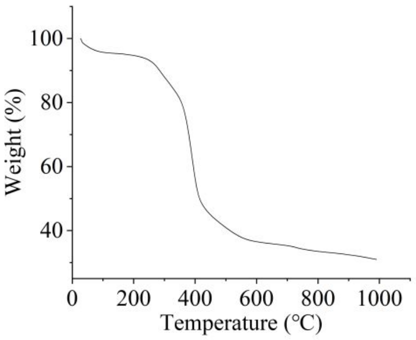

Figure S4. TGA analysis of SDMP.

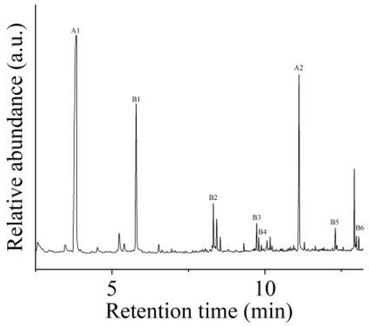

Figure S5. Characteristic peak of SDMP by Py-GCMS.

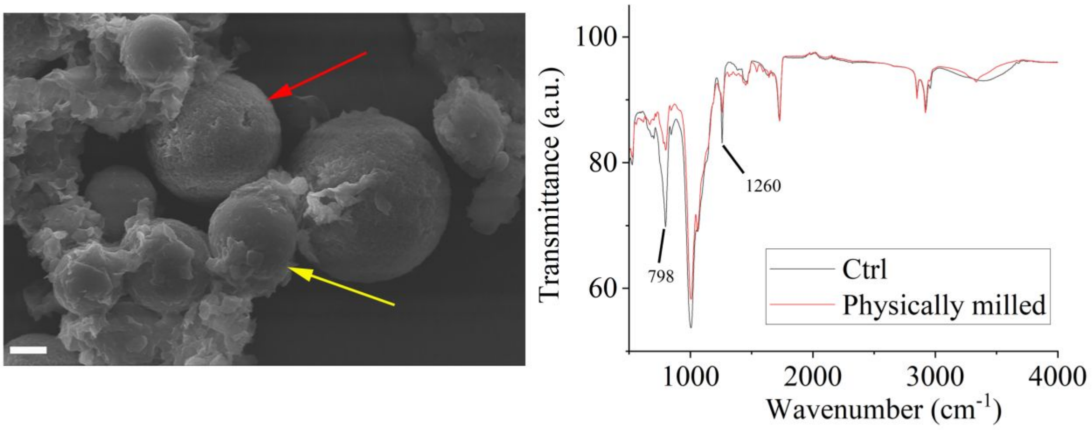

Figure S6. Morphology (left) and surface chemistry (right) of SDMP. The red arrow and the yellow arrow denote EMA-MMA (rough-surfaced microbead) and HSP (smooth-surfaced microbead), respectively. Scale bar: 2 µm.

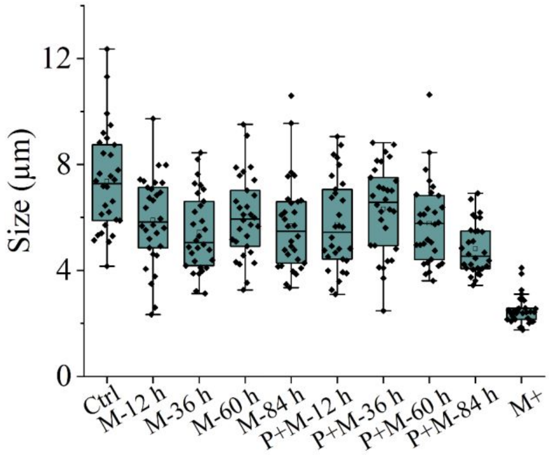

Figure S7. Size of MPs after degradation.

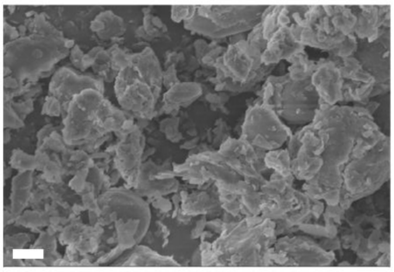

Figure S8. Morphology of the intense mechanically milled SDMP (M+ group). Scale bar: 2 μm.

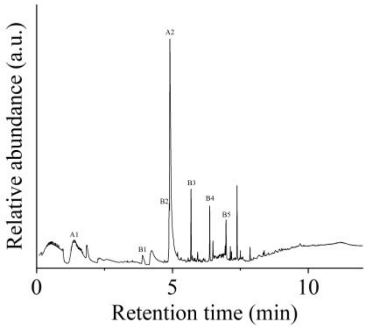

Figure S9. Characteristic peak of associated chemicals on SDMP.

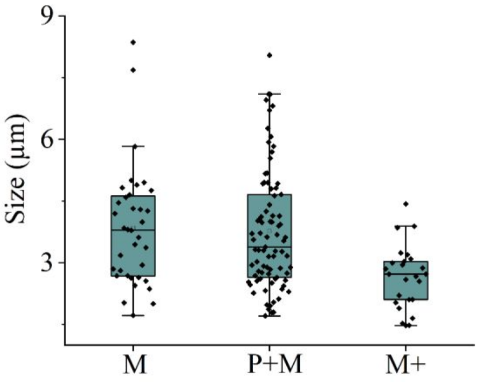

Figure S10. Size of ingested MPs.

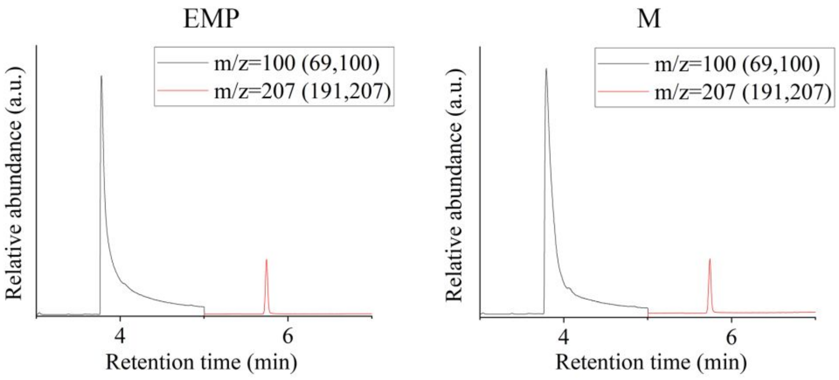

Figure S11. Difference between the characteristic peaks of SDMP before and after cellular uptake.

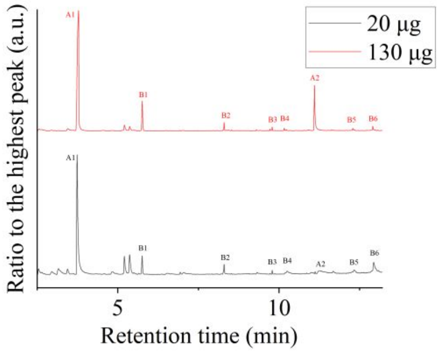

Figure S12. Characteristic peaks of SDMP with different concentrations.

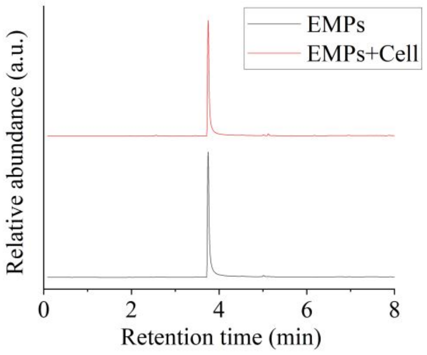

Figure S13. Effect of cell matrix on the intensity of the quantitative peak (m/z=100).

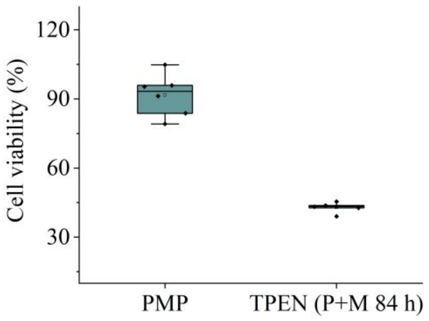

Figure S14. Cell viability in the presence of primary SDMP or TPEN chelators.

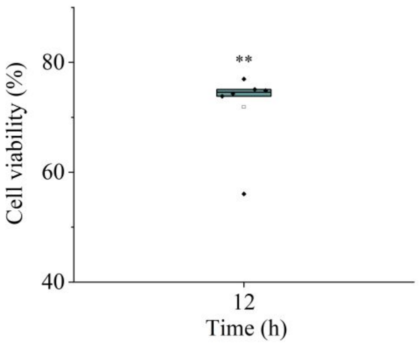

Figure S15. Cell viability influenced by the presence of intense mechanically milled SDMP.

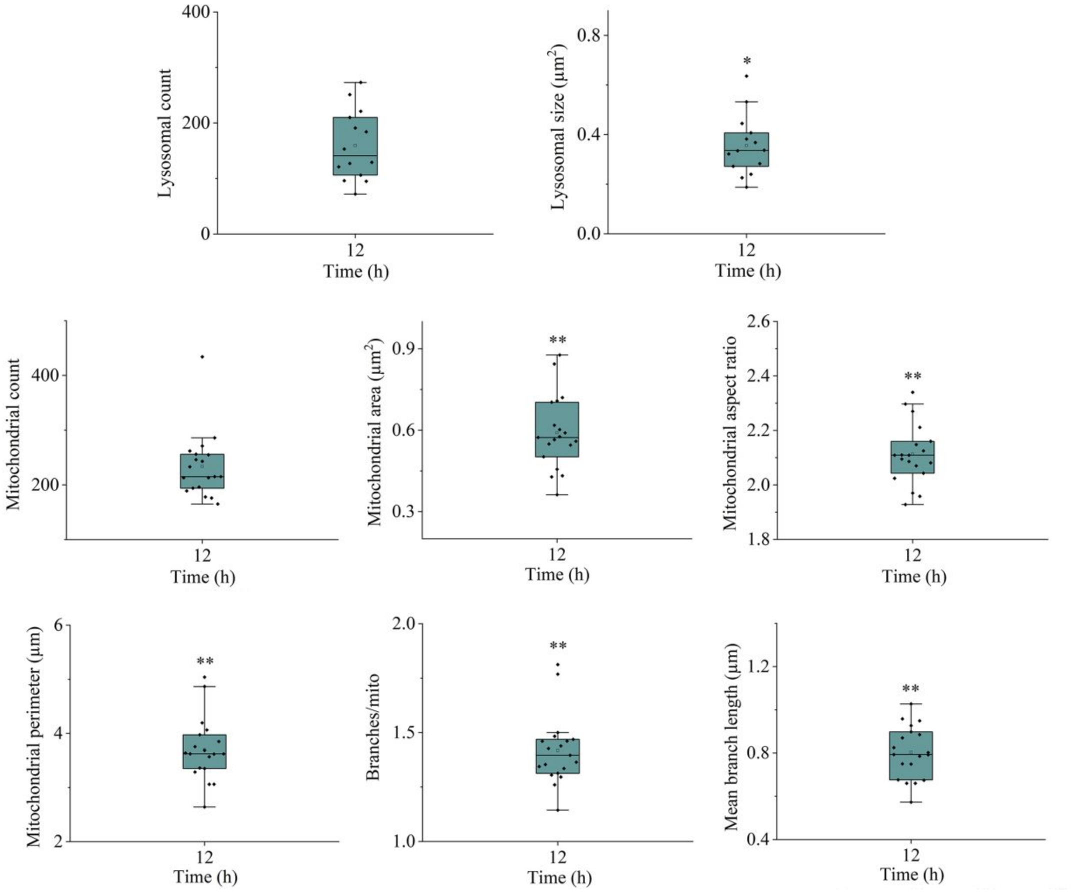

Figure S16. Subcellular effect of the intense mechanically milled SDMP.

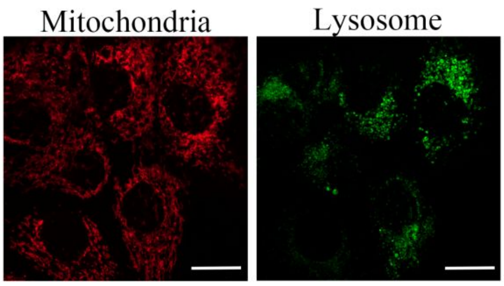

Figure S17. Subcellular biomarkers of cells in the control group. Scale bar: 20 μm.

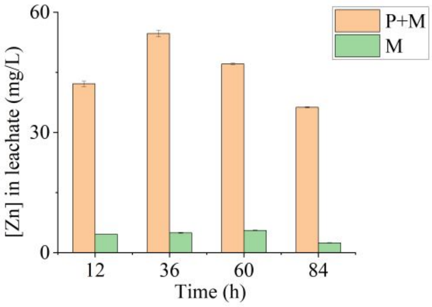

Figure S18. Concentration of total Zn in leachate of SDMP.

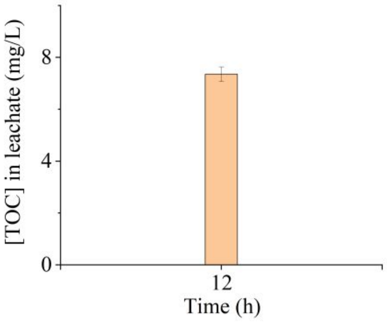

Figure S19. Concentration of TOC in 12 h-leachate of intense mechanically milled SDMP.

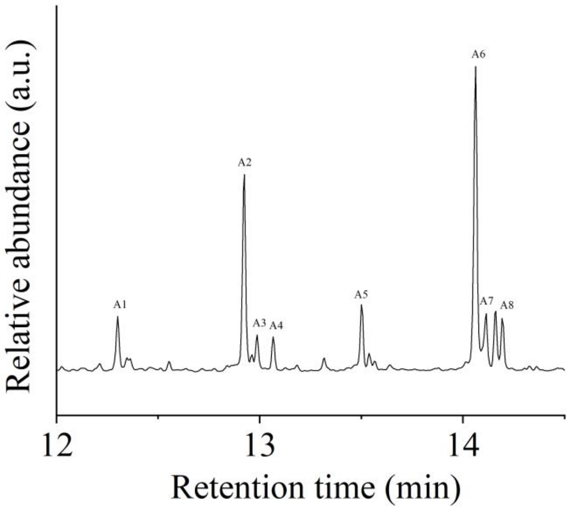

Figure S20. Characteristic peak denoting paraffin in SDMP.

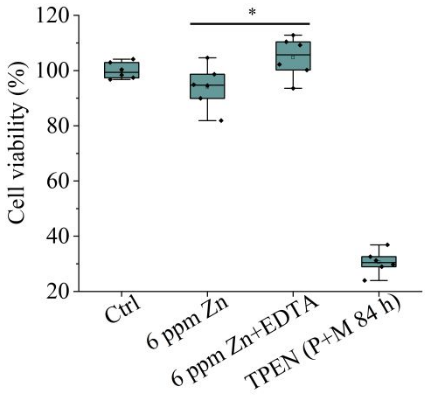

Figure S21. Effect of ionic Zn on cell viability.

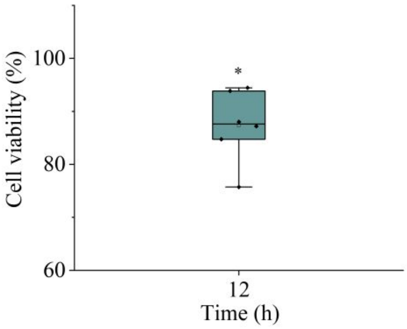

Figure S22. Effect of 12 h-leachate from the intense mechanically milled SDMP on cell viability.

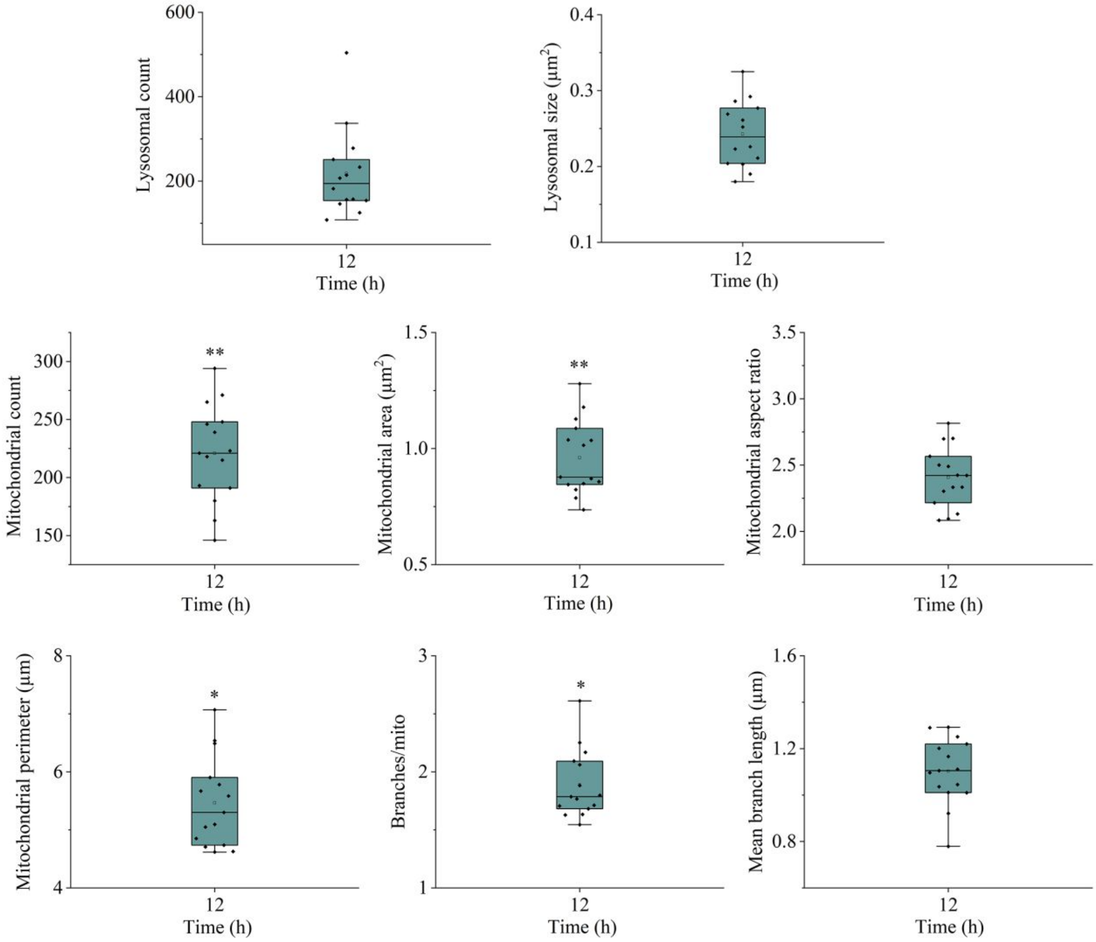

Figure S23. Subcellular effect of the 12 h-leachate from the intense mechanically milled SDMP.
